# Cardiorespiratory Fitness, Body Fat, and Physical Activity as Predictors of Life Satisfaction in Teachers and Preschool Education Students: The Mediating Role of Self-Rated Health

**DOI:** 10.3390/ijerph23030335

**Published:** 2026-03-07

**Authors:** Ivana Nikolić, Snježana Mraković, Marko Badrić

**Affiliations:** Faculty of Teacher Education, University of Zagreb, 10000 Zagreb, Croatia; ivana.nikolic@ufzg.hr (I.N.); marko.badric@gmail.com (M.B.)

**Keywords:** body composition, cardiorespiratory fitness (VO_2_max), life satisfaction, physical activity, preschool teachers, self-rated health, teacher education

## Abstract

**Highlights:**

**Public health relevance—How does this work relate to a public health issue?**

- Examines life satisfaction and self-rated health as widely recognized public health indicators of future health-related behaviors in students preparing for teacher and preschool education professions.
- Focuses on future educators who represent an important group for shaping children’s health behaviors and lifestyle patterns in early childhood.

**Public health significance—Why is this work of significance to public health?**

- Shows that self-rated health explains substantial variance in life satisfaction (17.2%), confirming its central role as a practical public health indicator.
- Demonstrates that overall and moderate physical activity are associated with life satisfaction with small but statistically significant effects (ΔR^2^ ≈ 2%), supporting public health recommendations to promote physical activity levels for mental well-being.

**Public health implications—What are the key implications or messages for practitioners, policymakers, and/or researchers in public health?**

- Suggests potential benefits from strengthening health promotion, physical activity education, and health psychology content in teacher and preschool education programs.
- Indicates that improving health perception and physical activity habits in future educators may contribute to healthier environments for children and support long-term public health goals.

**Abstract:**

**Objectives:** This study aimed to examine the associations of physiological (VO_2_max), morphological (body fat percentage), and behavioral factors (physical activity levels) with life satisfaction among teacher education and preschool education students, with a particular focus on self-rated health as a potential statistical mediator. **Methods:** The sample consisted of 228 students (95% female; mean age = 21.96 ± 4.24 years). Life satisfaction was assessed using the Satisfaction with Life Scale (SWLS), self-rated health (SRH) with a single-item measure of general health, physical activity (PA) with the IPAQ-SF questionnaire, cardiorespiratory fitness (VO_2_max estimate) with the 20 m shuttle run test, and body fat percentage (BF%) with a bioelectrical impedance analyzer. Data were analyzed using hierarchical regression and mediation models with the PROCESS macro (Model 4). **Results:** SRH accounted for 17.2% of the variance in life satisfaction, emerging as the strongest correlate relative to physiological and morphological indicators. In the primary regression model, total PA accounted for an additional 2.3% of explained variance, whereas in a secondary parallel model, moderate PA accounted for 2.2%. Vigorous PA was not a significant correlate. Mediation analyses indicated that VO_2_max was not directly associated with life satisfaction but showed a statistically significant indirect association through SRH. No significant indirect associations were found for BF%, individual activity intensities, or total PA. **Conclusions**: The results highlight the relevance of perceptual and behavioral health indicators in understanding the relationships among PA, physical fitness, and psychological well-being in this student population. The findings provide preliminary insight into how multiple health-related dimensions may relate to life satisfaction, underscoring the need for longitudinal research before more confident interpretations of practical relevance can be made.

## 1. Introduction

The psychological well-being of students is an important indicator of quality of life, mental health, and academic functioning. Previous studies have reported a high prevalence of depressive symptoms, anxiety, and reduced life satisfaction among student populations [1,2], with the university period representing a particularly sensitive developmental stage. Students of teacher education and preschool education face both academic and professional demands, and in their future roles, they are expected to act as active promoters of children’s health and physical activity. This further emphasizes the importance of maintaining their own well-being [3].

Physical activity (PA) has been consistently associated with higher subjective well-being and better mental health outcomes [4,5]. According to the World Health Organization [6], regular moderate-to-vigorous PA contributes to the prevention of chronic diseases and the promotion of mental health. The biopsychosocial model [7] and contemporary transdisciplinary approaches [8] emphasize that the effects of PA should be considered in interaction with physiological and psychosocial mechanisms.

Among physiological indicators, cardiorespiratory fitness (VO_2_max) has been identified as a strong predictor of cardiovascular health and mortality, as well as of psychological well-being in young adults [9,10]. Unfavorable body composition and elevated body mass index (BMI) are associated with an increased risk of metabolic disorders, lower life satisfaction, and greater incidence of depressive symptoms [11,12]. While some studies have demonstrated associations between higher VO_2_max and better well-being or lower emotional difficulties [13,14], findings are not always consistent, and other factors, such as muscular strength and coordination, may also play a role [15].

Perceptual factors, such as self-rated health (SRH), have emerged as robust predictors of mortality and mental health [16,17]. Furthermore, SRH has been shown to mediate the relationship between PA and subjective well-being [18,19]. However, prior research has predominantly focused on behavioral and perceptual indicators, often relying on self-reported measures, without explicitly differentiating physiological capacity from behavioral engagement in PA.

Although associations between PA and life satisfaction, as well as the mediating role of SRH, have been documented, fewer studies have simultaneously integrated objectively assessed physiological capacity (VO_2_max), morphological characteristics (body fat percentage), behavioral engagement in PA, and subjective health evaluation within a single explanatory framework. Consequently, it remains unclear whether life satisfaction is primarily associated with physiological fitness per se, with subjective health appraisals, or with the motivational and experiential dimensions of PA engagement.

Within the biopsychosocial framework [7], it is therefore essential to distinguish between physiological capacity, health-related behaviors, and subjective health evaluations. Cardiorespiratory fitness (VO_2_max) may be conceptualized as an indicator of functional physiological capacity rather than a consciously perceived attribute. Individuals do not directly perceive maximal oxygen uptake as a discrete physiological parameter; instead, they interpret its functional consequences, such as vitality, endurance, and resistance to fatigue. These experiential manifestations may be cognitively integrated into global health evaluations, such as SRH, which can function as a perceptual bridge, linking physiological capacity to psychological well-being.

In contrast, PA constitutes not only a determinant of physiological fitness but also a psychologically meaningful behavior. According to Self-Determination Theory [20], engagement in PA may directly enhance life satisfaction through the satisfaction of basic psychological needs for autonomy, competence, and relatedness. These motivational and affective processes may contribute to well-being independently of physiological fitness, suggesting that behavioral engagement and physiological capacity operate through partially distinct pathways.

The present study, therefore, aimed to investigate the contribution of these factors to life satisfaction, with a particular focus on the mediating role of SRH. We hypothesized that (1) higher VO_2_max and lower body fat percentage would be associated with higher life satisfaction, (2) higher levels of PA would be positively associated with life satisfaction, and (3) SRH would statistically mediate the associations between physiological indicators and life satisfaction.

## 2. Materials and Methods

### 2.1. Sample of Participants

This study was conducted on a convenience sample of students enrolled in teacher education and preschool education programs in the city of Čakovec (Faculty of Teacher Education, University of Zagreb, Čakovec Department). A total of 228 participants took part, with a mean age of 21.96 ± 4.24 years. From the initial sample of 244 participants, 16 were excluded due to incomplete questionnaires, chronic illnesses or injuries preventing participation in the shuttle run test, or lack of informed consent. The sample was predominantly female (95%), which reflects the typical gender distribution in teacher and preschool education programs and is considered representative of this specific professional context.

### 2.2. Sample of Variables

The criterion variable in this study was life satisfaction, assessed with the Satisfaction with Life Scale (SWLS) [21], in its Croatian adaptation. The scale consists of 5 items rated on a 7-point Likert scale (1 = strongly disagree to 7 = strongly agree) and measures the cognitive evaluation of one’s life. In this study, the scale demonstrated high reliability (Cronbach’s α = 0.87).

Self-rated health (SRH) was assessed with a single question: “How would you rate your health in recent times?” Responses ranged from 1 (very poor) to 5 (excellent). This single-item measure has been validated and shown to be a strong predictor of health outcomes such as mortality and chronic disease [16,22,23].

Maximal oxygen uptake (VO_2_max) was estimated using the adult version of the 20-m shuttle run test [24]. Participants ran back and forth between two lines placed 20 m apart while following standardized pre-recorded audio signals that dictated the running pace. The test commenced at 8.0 km/h and increased by 0.5 km/h every two minutes. The test was terminated when participants reached volitional exhaustion or failed on two consecutive occasions to reach the line before the audio signal. The speed corresponding to the final fully completed stage was used to estimate VO_2_max according to the regression equation [24]: VO_2_max (mL/kg/min) = 5.857 × speed (km/h) − 19.458.

Body fat percentage (BF%) was assessed using a Tanita RD-545 bioelectrical impedance analyzer (Tanita Corp., Tokyo, Japan; sourced from Tanita Europe B.V., Amsterdam, The Netherlands), which has demonstrated satisfactory metric characteristics. The use of Tanita bioelectrical impedance analysis for body composition assessment is supported by previous studies showing acceptable validity and reliability in youth and young adult populations, with body fat estimates comparable to criterion methods such as dual-energy X-ray absorptiometry, hydrodensitometry, and skinfold measurements, particularly at the group level [25,26,27]. Bioelectrical impedance analysis (BIA) measurements were conducted under standardized conditions to enhance reproducibility. Participants were instructed not to consume food, caloric beverages, and caffeine for at least 4 h prior to testing, abstain from alcohol for 24–48 h, and refrain from moderate-to-vigorous exercise for 12–24 h before assessment. Participants were advised to maintain normal hydration but avoid excessive fluid intake in the 2 h before testing and to void their bladder within 30 min before measurement. All assessments were performed in the morning hours in a temperature-controlled environment (20–25 °C), using the same calibrated device. Participants wore light clothing and removed shoes, socks, and metal accessories. They were positioned with the arms slightly abducted and legs apart, and electrode placement was standardized across all measurements. All participants were healthy at the time of testing.

Physical activity (PA) was assessed using the short form of the International Physical Activity Questionnaire (IPAQ-SF), which captures the frequency (days/week) and duration (minutes/day) of vigorous, moderate, and walking activities during the previous seven days. Data were processed according to the official IPAQ scoring guidelines. Only activity bouts lasting at least 10 min were included. Durations shorter than 10 min were recorded as zero. Daily activity exceeding 180 min in any intensity category was truncated to minimize implausible values. Weekly MET-minutes were calculated as MET × minutes/day × days/week (vigorous = 8.0 MET, moderate = 4.0 MET, walking = 3.3 MET), and total PA was computed as the sum of all intensity categories. The Croatian version of the IPAQ-SF has demonstrated satisfactory reliability [28]. In regression analyses, total PA (MET-min/week) was defined as the primary behavioral predictor, while intensity-specific variables were examined in supplementary parallel models.

### 2.3. Study Procedure

The study was conducted in November 2025 during classes in the course Physical Education, where standardized kinesiology tests are administered at the beginning and end of each academic year to monitor progress among adult students and to provide training in fitness assessments for working with children. Students anonymously completed questionnaires on PA, SRH, and life satisfaction via Google Forms after providing informed consent. Participation was voluntary and uncompensated, with no academic or financial incentives. The study was carried out in accordance with the ethical principles of the Declaration of Helsinki [29] and institutional research ethics guidelines.

### 2.4. Statistical Analysis

Statistical analyses were conducted using IBM SPSS Statistics for Windows, version 29.0 (IBM Corp., Armonk, NY, USA). The distributions of all variables were examined prior to modelling, and all variables were retained in their original metric to preserve interpretability. Before conducting the regression analyses, key OLS assumptions were evaluated, including linearity, homoscedasticity, normality of residuals, the absence of multicollinearity (VIF, Tolerance, Condition Index), and the absence of influential observations (Cook’s distance and leverage). No substantial violations were detected. Hierarchical regression models were then used to examine the incremental contribution of successive predictor blocks. Model 1 included demographic and subjective health indicators (age and SRH). Model 2 included the objective physiological indicators (VO_2_max and BF%). Model 3 further included the behavioral factor of PA. In accordance with the aims of the study, the model including total PA (MET-min/week) was defined as the primary regression model.

Given that total PA is mathematically derived from its intensity-specific components (vigorous, moderate, and walking MET-min/week) and these components are strongly intercorrelated, they were not entered simultaneously into the same regression equation. Instead, separate parallel models were estimated in which each intensity component was entered individually in place of the total PA. These intensity-specific models were treated as supplementary exploratory analyses and are presented in Supplemental Table S1; Table S2. Effect sizes (f^2^) were interpreted according to Cohen’s criteria, with values of 0.02 indicating a small effect, 0.15 a medium effect, and 0.35 a large effect [30]. A post hoc power analysis was conducted in G*Power 3.1.9.4 for the sample of 228 participants (α = 0.05) [31]. For Model 1, with an effect size of f^2^ = 0.208, statistical power was 0.999. For the primary Model 3 (total PA), with an effect size of f^2^ = 0.030, power was 0.699. Power for Model 2 was not separately estimated, as adding physiological indicators did not increase the explained variance (ΔR^2^ = 0.000; f^2^ = 0.000).

Mediation analyses were conducted using the PROCESS macro version 5.0 [32], Model 4, with 5000 bootstrap samples and 95% confidence intervals. The aim was to test whether SRH mediated the relationship between VO_2_max, BF%, PA, and SWLS. In all mediation models, age and the remaining two main predictors (depending on which predictor was entered as variable X) were included as covariates.

## 3. Results

Descriptive statistics for all study variables are presented in Table 1. Means, ranges, and standard deviations reflect expected values for a student population.

Table 2 presents the correlation coefficients for the study variables. SRH showed a statistically significant positive association with SWLS (*r* = 0.408, *p* < 0.001). TPA demonstrated a weak but significant positive relationship with SWLS (*r* = 0.184, *p* = 0.005). Age was negatively associated with SWLS (*r* = −0.132, *p* = 0.047). VO_2_max was significantly positively correlated with SRH (*r* = 0.217, *p* < 0.001), while BF% was negatively correlated with both VO_2_max (*r* = −0.446, *p* < 0.001) and SRH (*r* = −0.201, *p* = 0.002). The intensity-specific components of PA (VPA, MPA, WAL) showed a similar pattern of associations with SWLS as TPA, but none of them demonstrated stronger associations than TPA overall. No other correlations were statistically significant.

In the first model (Table 3), age and self-rated health together explained 17.2% of the variance in life satisfaction (R^2^ = 0.172, *p* < 0.001). Almost the entire contribution was attributable to self-rated health, which emerged as a significant correlate (β = 0.397, *p* < 0.001), while age was not significant (*p* = 0.203). Adding VO_2_max and body fat percentage in the second model did not change the explained variance (ΔR^2^ = 0.000; f^2^ = 0.000), indicating a negligible effect size, with neither physiological indicator reaching significance (*p* > 0.05).

In the primary Model 3, total PA accounted for an additional 2.3% of the variance in life satisfaction (ΔR^2^ = 0.023; f^2^ = 0.030) and was significantly associated with life satisfaction (β = 0.153, *p* = 0.014). A parallel model including moderate PA instead of total PA accounted for an additional 2.2% of the variance (ΔR^2^ = 0.022; f^2^ = 0.027), with moderate PA also emerging as a significant predictor (β = 0.148, *p* = 0.017). Additional models including vigorous PA and walking did not reach statistical significance (results are presented in Tables S1 and S2).

**Figure 1:**
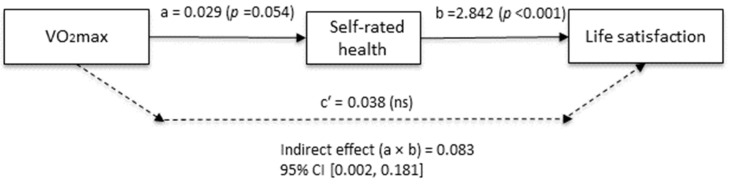
Mediation model of VO_2_max and life satisfaction via self-rated health.

VO_2_max was indirectly associated with life satisfaction through self-rated health (B = 0.083; 95% CI [0.002–0.181]), while the direct effect was not statistically significant (c′ = 0.038, *p* = 0.707) (Figure 1). Body fat percentage did not show significant direct or indirect associations with life satisfaction. In contrast, total PA (*p* = 0.014) and moderate PA (*p* = 0.017) demonstrated small but statistically significant direct associations with life satisfaction, whereas their indirect effects were not significant (Table 4). Vigorous PA was not significantly associated with life satisfaction (results are presented in Table S3).

## 4. Discussion

The present findings support a differentiated pattern of associations between physiological, morphological, and behavioral indicators and life satisfaction among teacher education and preschool education students. Cardiorespiratory fitness (VO_2_max) was associated with life satisfaction in a pattern consistent with statistical mediation through self-rated health (SRH), whereas moderate and total physical activity (PA) demonstrated small but statistically significant direct associations. Body fat percentage (BF%) was not significantly related to life satisfaction. Given the cross-sectional design, these results reflect concurrent associations and do not permit conclusions regarding temporal ordering. Overall, the findings underscore the relevance of subjective health appraisal as a potential explanatory pathway linking physiological capacity to psychological well-being, in line with prior research emphasizing perceptual processes [18,19].

### 4.1. VO_2_max and Life Satisfaction: The Role of Self-Rated Health

In this study, VO_2_max, conceptualized as an indicator of physiological capacity, was not directly associated with life satisfaction. Instead, the pattern of results was statistically consistent with an indirect association through self-rated health. Higher VO_2_max was associated with more positive health evaluations, which were in turn associated with greater life satisfaction. This pattern aligns with the biopsychosocial model [7] which proposes that physiological indicators may acquire psychological significance through subjective interpretation.

Previous studies have documented direct associations between cardiorespiratory fitness and mental health in adolescents and young adults [9,13,14], generally without accounting for potential mediating mechanisms. Partial support for a mediated pathway is provided by research showing that self-efficacy mediated the relationship between physical fitness and stress resilience [33]. Extending this line of work, the present findings suggest that, in this student population, the association between cardiorespiratory fitness and life satisfaction may be indirectly reflected through self-rated health, underscoring the role of subjective health appraisal in linking physiological capacity to psychological well-being. It should be noted that VO_2_max in this study was estimated using the 20 m shuttle run test, which, although widely validated and practical for field-based assessments [34,35], remains an indirect estimate compared to laboratory-based gas analysis. Future research employing direct measurement of oxygen uptake may help reduce measurement error in the estimation of aerobic capacity and further clarify its relationship with psychological well-being. Given that PA and VO_2_max were moderately correlated in this sample, small direct effects of VO_2_max cannot be ruled out. Alternative conceptual models, including reverse mediation or bidirectional associations, also remain plausible given the cross-sectional design.

### 4.2. Physical Activity and Life Satisfaction

Total PA accounted for 2.3% of the variance in life satisfaction in the primary regression model (f^2^ = 0.030), while moderate PA explained 2.2% in a parallel model (f^2^ = 0.027). Although statistically significant, these effects were small in magnitude according to Cohen’s criteria and should therefore be interpreted cautiously. Accordingly, the practical relevance of these associations appears modest. The observed associations are consistent with meta-analytic evidence reporting small-to-moderate positive relationships between PA and subjective well-being [4,17]. Similar patterns have been documented in university student populations, where PA has been positively associated with quality of life and psychological outcomes [36,37,38]. Within this context, PA may be understood as one of several behavioral factors concurrently associated with life satisfaction in this population.

Previous research has suggested that the association between PA and well-being may be partially explained by psychological mechanisms, including psychological need satisfaction [39,40] and self-efficacy [41]. In the present study, total and moderate PA demonstrated direct associations with life satisfaction. This pattern may reflect immediate experiential and social dimensions of engagement in PA, such as enjoyment, social interaction, and stress reduction. Such an interpretation is consistent with self-determination theory [20], which suggests that activities supporting autonomy, competence, and relatedness are positively associated with subjective well-being.

It should be noted that PA was assessed using the IPAQ-SF, a self-report instrument that may overestimate activity levels compared with device-based assessments. Future research employing objective measures may help refine the magnitude of these associations. Although PA and VO_2_max are generally positively correlated [42], the present findings suggest that their relationships with life satisfaction may reflect partially distinct pathways, with PA demonstrating a direct association and cardiorespiratory fitness showing an indirect association via self-rated health.

### 4.3. Body Fat Percentage and Life Satisfaction

In this study, body fat percentage did not show a significant association with life satisfaction, either in correlation analyses or mediation models. This finding is consistent with research on young adults reporting weak or inconsistent relationships between body mass index (BMI), body fat percentage, and psychological outcomes [43,44,45]. These results are consistent with evidence suggesting that objective morphological indicators are less relevant for subjective well-being outcomes than perceptual mechanisms. Specifically, previous research has demonstrated that subjective evaluations of health and body image are more strongly associated with life satisfaction than objectively assessed body composition [46]. Furthermore, research has indicated that positive body-related perceptions predict life satisfaction even after controlling for BMI and other individual characteristics, thereby further underscoring the limited role of objective body composition in explaining life satisfaction [47].

Given the modest statistical power for detecting small effects in incremental models, the non-significant association between body fat percentage and life satisfaction should be interpreted with caution. Although the direction of effects was consistent with prior research, the possibility of Type II error cannot be fully excluded, and future studies with larger samples may be better positioned to detect subtle relationships.

### 4.4. Practical Implications

Although modest in magnitude, the findings of this study offer considerations for understanding well-being among teacher education and preschool education students within educational contexts. The differentiated pattern of associations observed between physiological capacity, behavioral engagement in PA, and subjective health appraisal suggests that multiple dimensions of health may be relevant when reflecting on students’ life satisfaction.

In educational environments preparing future professionals who are expected to promote health and active lifestyles, attention to how PA experiences and health perceptions are situated within the broader study context may be conceptually meaningful. Established motivational frameworks emphasizing the roles of autonomy, competence, and self-efficacy in sustaining health-related behaviors [20,48] provide a theoretical lens through which these findings can be interpreted.

Given the cross-sectional design and small effect sizes, these implications should be interpreted cautiously. Further longitudinal and experimental research would be necessary to clarify how these health-related dimensions interact over time within student populations.

### 4.5. Limitations and Future Research

This study has several limitations that should be considered when interpreting the findings. First, the cross-sectional design does not allow conclusions regarding the directionality of the observed associations. Although the analyses were statistically consistent with mediation patterns, causal relationships cannot be established.

Second, the predominantly female composition of the sample (95%), reflecting the gender structure typical of teacher education and preschool education programs, limits the generalizability of the findings to male students or more gender-balanced populations.

Third, the use of self-reported measures for PA and self-rated health may introduce recall or social desirability bias. In addition, cardiorespiratory fitness was estimated using a validated field-based assessment (20 m shuttle run test), which, although appropriate for population-level research, is less precise than laboratory-based gas analysis.

Future research employing longitudinal or experimental designs would be valuable for examining how these associations develop over time. Studies including more gender-balanced samples would further strengthen the generalizability of the findings. Exploring additional psychological and social factors, such as social support, body image, or emotional regulation, may also contribute to a more comprehensive understanding of the associations among physiological, behavioral, and perceptual factors and life satisfaction.

## 5. Conclusions

In the sample of teacher education and preschool education students, self-rated health emerged as the strongest correlate of life satisfaction, while total and moderate physical activity demonstrated small but statistically significant associations. Cardiorespiratory fitness showed an indirect association with life satisfaction through self-rated health, a pattern statistically consistent with mediation rather than causal inference.

Collectively, the findings provide preliminary insight into how perceptual, behavioral, and physiological indicators may relate to well-being in this population. The differentiated associations observed across these domains highlight the relevance of considering both objective health-related characteristics and subjective health appraisals when examining life satisfaction. Further longitudinal research will be essential for clarifying the direction and stability of these relationships over time.

## Figures and Tables

**Table 1:** Descriptive statistics for predictor and criterion variables.

Variable	Min	Max	M	SD	Skew	Kurt
Life satisfaction (SWLS)	9	35	25.26	5.60	−0.55	−0.16
Age (years)	18	41	21.96	4.24	2.28	4.99
Self-rated health (SRH)	1	5	3.67	0.78	−0.37	0.16
Body fat percentage (BF%)	6	56.80	34.27	8.91	−0.11	0.54
VO_2_max (mL/kg/min)	33.26	53.75	36.89	3.79	1.56	3.49
Vigorous PA (MET-min/week)	0	3000	807.41	1025.71	1.08	−0.13
Moderate PA (MET-min/week)	0	3000	771.68	747.22	0.96	0.55
Walking (MET-min/week)	0	3000	929.67	595.23	0.93	0.16
Total PA (MET-min/week)	33	10,053	2576.87	1682.52	0.89	1.02

**Table 2:** Correlation analysis of predictor and criterion variables.

	SWLS	AGE	SRH	VO_2_max	BF%	VPA	MPA	WAL	TPA
SWLS	1								
AGE	−0.132 *	1							
SRH	0.408 **	−0.135 *	1						
VO_2_max	0.090	−0.144 *	0.217 **	1					
BF%	−0.074	0.137 *	−0.201 **	−0.446 **	1				
VPA	0.143 *	−0.077	0.152 *	0.186 **	−0.038	1			
MPA	0.184 **	−0.119	0.075	0.096	0.016	0.346 **	1		
WAL	−0.001	−0.007	0.010	−0.064	−0.051	−0.051	−0.106	1	
TPA	0.184 **	−0.111	−0.016	0.152 *	−0.016	0.847 **	0.656 **	0.282 **	1

Note. * *p* < 0.05, ** *p* < 0.01 (2-tailed); SWLS = Satisfaction with Life Scale, AGE = age, SRH = self-rated health, VO_2_max = maximal oxygen uptake (mL/kg/min), BF% = body fat percentage, VPA = vigorous PA (MET-min/wk), MPA = moderate PA (MET-min/wk), WAL = walking (MET-min/wk), TPA = total PA (MET-min/wk).

**Table 3:** Hierarchical regression analysis predicting life satisfaction.

Model	Predictor	β (Beta)	t	*p*	R^2^	ΔR^2^
1	Self-rated health	0.397	6.473	<0.001	0.172	–
	Age (years)	−0.078	−1.277	0.203		
2	Self-rated health	0.400	6.326	<0.001	0.172	0.000
	Age (years)	−0.080	−1.291	0.198		
	Body Fat Percentage	0.018	0.255	0.799		
	VO_2_max	0.000	−0.002	0.998		
3	Self-rated health	0.394	6.299	<0.001	0.195	0.023
	Age (years)	−0.066	−1.074	0.284		
	Body Fat Percentage	0.005	0.075	0.940		
	VO_2_max	−0.026	−0.376	0.707		
	Total PA (MET-min/week)	0.153	2.480	0.014		
3a	Self-rated health	0.393	6.276	<0.001	0.194	0.022
	Age (years)	−0.064	−1.040	0.299		
	Body Fat Percentage	0.003	0.050	0.960		
	VO_2_max	−0.018	−0.261	0.794		
	Moderate PA (MET-min/week)	0.148	2.412	0.017		

Note. β = standardized regression coefficient; R^2^ = coefficient of determination; ΔR^2^ = change in explained variance. Physical activity (PA) components were analyzed separately due to multicollinearity.

**Table 4:** Mediation of physical fitness and activity indicators on life satisfaction via self-rated health.

Predictor	Path a (X→M)	Path b (M→Y)	Direct Effect c′ (X→Y)	Indirect Effect a × b	BootSE	BootLLCI	BootULCI
Body Fat Percentage	−0.011 (*p* = 0.091)	2.842 (*p* < 0.001)	0.003 (*p* = 0.940)	−0.031	0.019	−0.071	0.006
VO_2_max	0.029 (*p* = 0.054)	2.842 (*p* < 0.001)	0.038 (*p* = 0.707)	0.083	0.045	0.002	0.181
Total PA	0.000 (*p* = 0.569)	2.842 (*p* < 0.001)	0.001 (*p* = 0.014)	0.000	0.000	−0.000	0.000
Moderate PA	0.000 (*p* = 0.493)	2.835 (*p* < 0.001)	0.001 (*p* = 0.017)	0.000	0.000	−0.000	0.001

Note. Path a = effect of X on the mediator (M); Path b = effect of M on Y controlling for X; c′ = direct effect of X on Y controlling for M; indirect effects (a × b) are based on 5000 bootstrap samples; statistical significance of the indirect effect is determined by the 95% CI not including zero. All models control for the remaining two predictors and age.

## Data Availability

The data from this article will be made available by the authors upon reasonable request.

## Supplementary Materials

The following supporting information can be downloaded at: https://www.mdpi.com/article/10.3390/ijerph23030335/s1, Table S1: Hierarchical regression models with vigorous physical activity as predictor; Table S2: Hierarchical regression models with walking as predictor; Table S3: Mediation analysis with vigorous physical activity as predictor—Model 4/ Note. LLCI and ULCI refer to the lower and upper bounds of the 95% confidence interval based on 5000 bootstrap samples. All analyses were conducted in SPSS using the PROCESS macro (version 5.0). Covariates included all other predictor variables and age (Age-years)
