# Supplementary material for: Cardiorespiratory Fitness, Body Fat, and Physical Activity as Predictors of Life Satisfaction in Teachers and Preschool Education Students: The Mediating Role of Self-Rated Health
Source: Int J Environ Res Public Health. 2026 Mar 7;23(3):335. doi: 10.3390/ijerph23030335 (PMC13026139; doi:10.3390/ijerph23030335)
Supplement: Supplementary file 1 [file ijerph-23-00335-s001.zip › ijerph-4130742-supplementary.pdf]

International Journal of
Environmental Research and Public Health

MDPI

Article

Supplementary Materials

Additional hierarchical regression models including vigorous physical activity (MET-min/week) and walking (MET-min/week) were tested as predictors of life satisfaction. Full results of these models are presented in Tables S1 and S2.

Supplement S.1

For vigorous physical activity, inclusion in the final model resulted in a negligible increase in explained variance ($\Delta R^2 = .009$; $f^2 = 0.011$, very small effect), and the predictor was not statistically significant ($\beta = .097$, $p = .120$). Self-rated health remained a strong and significant predictor ($\beta = .394$, $p < .001$).

Table S1. Hierarchical regression models with vigorous physical activity as predictor.

Model	Predictor	β (Beta)	t	*p*	R^2	ΔR^2
1	Self-rated health	0.397	6.473	<.001	0.172	–
	Age (years)	-0.078	-1.277	0.203		
2	Self-rated health	0.4	6.326	<.001	0.172	0.0
	Age (years)	-0.08	-1.291	0.198		
	Body Fat Percentage	0.018	0.255	0.799		
	VO_2max	0.0	-0.002	0.998		
3	Self-rated health	0.394	6.237	<.001	0.181	0.009
	Age (years)	-0.077	-1.236	0.218		
	Body Fat Percentage	0.011	0.161	0.872		
	VO_2max	-0.018	-0.264	0.792		
	Vigorous PA (MET-min/week)	0.097	1.559	0.12		

Supplement S.2

For walking, inclusion in the final model resulted in a negligible increase in explained variance ($\Delta R^2 = .001$; $f^2 = 0.001$, negligible effect), and the predictor was not statistically significant ($\beta = .018$, $p = .765$). Self-rated health continued to show a strong and significant contribution ($\beta = .401$, $p < .001$).

Table S2. Hierarchical regression models with walking as predictor.

Model	Predictor	β (Beta)	t	*p*	R^2	ΔR^2
1	Self-rated health	0.397	6.473	<.001	0.172	–
	Age (years)	-0.078	-1.277	0.203		
2	Self-rated health	0.4	6.326	<.001	0.172	0.0
	Age (years)	-0.08	-1.291	0.198		
	Body Fat Percentage	0.018	0.255	0.799		
	VO_2max	0.0	-0.002	0.998		
3	Self-rated health	0.401	6.319	<.001	0.173	0.001
	Age (years)	-0.08	-1.284	0.2		
	Body Fat Percentage	0.018	0.259	0.795		
	VO_2max	0.001	0.015	0.988		
	Walking (MET-min/week)	0.018	0.299	0.765		

Supplement S.3

Mediation analysis with vigorous physical activity as predictor - Model 4 is presented in Table S3.

Table S3. Mediation analysis with vigorous physical activity as predictor – Model 4.

Effect	Coefficient	*p*	BootSE	BootLLCI	BootULCI
Path a (X → M)	0.0000	.355	–	−0.0001	0.0001
Path b (M → Y)	2.8409	< .001	–	1.9432	3.7386
Direct effect c′ (X → Y)	0.0005	.120	–	−0.0001	0.0012
Indirect effect (a × b)	0.0001	–	0.0002	−0.0002	0.0004

Note. LLCI and ULCI refer to the lower and upper bounds of the 95% confidence interval based on 5,000 bootstrap samples. All analyses were conducted in SPSS using the PROCESS macro (version 5.0). Covariates included all other predictor variables and age (Age-years).

https://doi.org/10.3390/ijerph23030335
